# Advanced Triboelectric Nanogenerators for Smart Devices and Emerging Technologies: A Review

**DOI:** 10.3390/mi16111203

**Published:** 2025-10-23

**Authors:** Van-Long Trinh, Chen-Kuei Chung

**Affiliations:** 1 Faculty of Mechanical Engineering, School of Mechanical and Automotive Engineering, Hanoi University of Industry, 298 Caudien Street, Hanoi 100000, Vietnam; longtv@haui.edu.vn; 2 Department of Mechanical Engineering, National Cheng Kung University, Tainan 701, Taiwan

**Keywords:** triboelectric nanogenerator, smart devices, emerging technologies, sustainable energy, renewable energy

## Abstract

Smart devices and emerging technologies are highly popular devices and technologies that considerably improve our daily living by reducing or replacing human workforces, treating disease, monitoring healthcare, enhancing service performance, improving quality, and protecting the natural environment, and promoting non-gas emissions, sustainable working, green technologies, and renewable energy. Triboelectric nanogenerators (TENGs) have recently emerged as a type of advanced energy harvesting technology that is simple, green, renewable, flexible, and endurable as an energy resource. High-performance TENGs, denoted as advanced TENGs, have potential for use in many practical applications such as in self-powered sensors and sources, portable electric devices, power grid penetration, monitoring manufacturing processes for quality control, and in medical and healthcare applications that meet the criteria for smart devices and emerging technologies. Advanced TENGs are used as highly efficient energy harvesters that can convert many types of wasted mechanical energy into the electric energy used in a range of practical applications in our daily lives. This article reviews recently advanced TENGs and their potential for use with smart devices and emerging technology applications. The work encourages and strengthens motivation to develop new smart devices and emerging technologies to serve us in many fields of our daily living. When TENGs are introduced into smart devices and emerging technologies, they can be applied in a variety of practical applications such as the food processing industry, information and communication technology, agriculture, construction, transportation, marine technology, the energy sector, mechanical processing, manufacturing, self-powered sensors, Industry 4.0, drug safety, and robotics due to their sustainable and renewable energy, light weight, cost effectiveness, flexibility, and self-powered portable energy sources. Their advantages, disadvantages, and solutions are also discussed for further research.

## 1. Introduction

**Figure 1:**
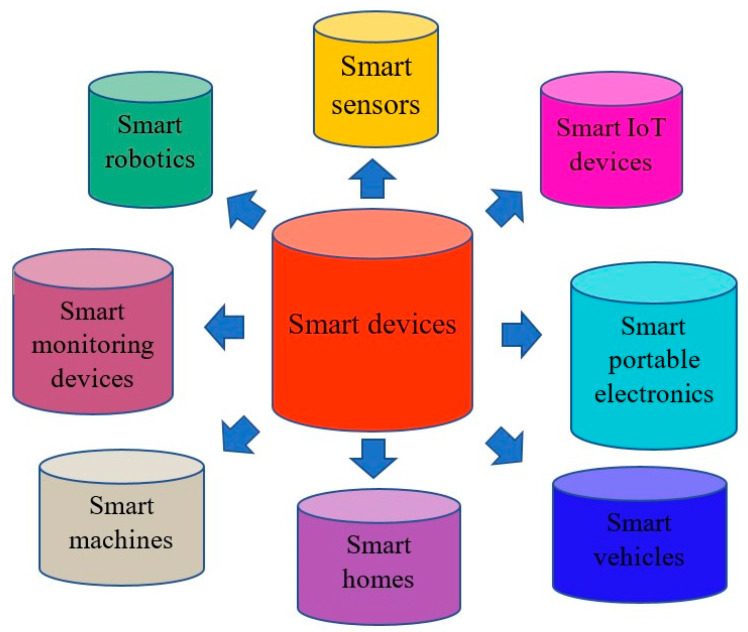
The smart devices developed for use in daily living: smart sensors, smart homes, smart monitoring devices, smart portable electronics, smart vehicles, smart machines, smart IoT devices, and smart robotics.

**Figure 2:**
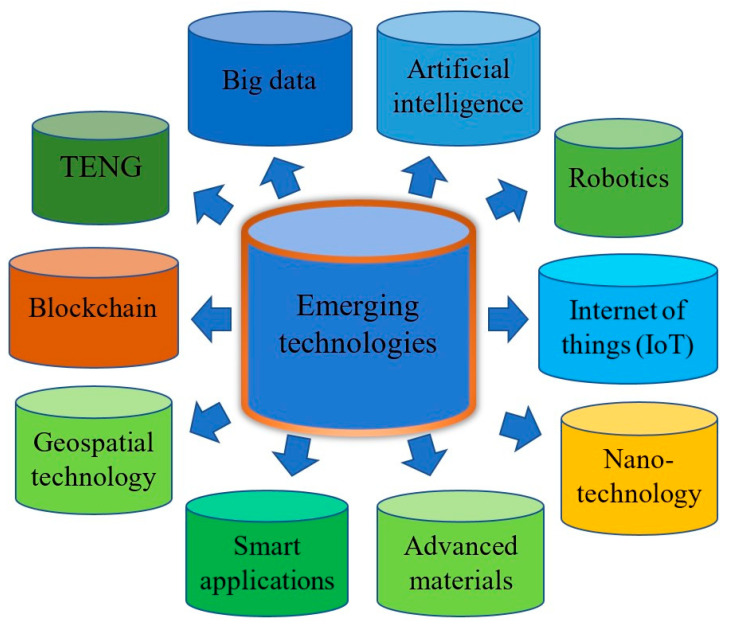
Successful emerging technologies that have been developed for practical applications: triboelectric nanogenerators, geospatial technology, artificial intelligence, big data, robotics, smart applications, blockchain, additive manufacturing, advanced materials, Internet of Things (IoT), and nanotechnology.

**Figure 3:**
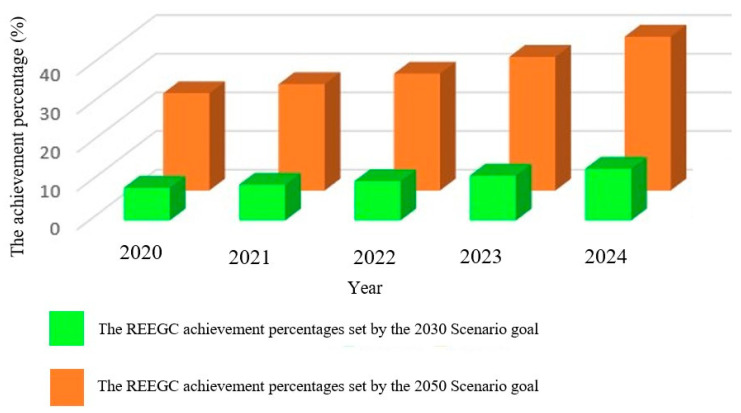
The percentages of REEGC achievements from 2020 to 2024 in comparison to the goals set by the 2030 and 2050 Scenarios for lowering global temperature of 1.5 °C.

Smart devices (SDs) and emerging technologies (ETs) are representations of the advancement of science, techniques, and technology that have brought many benefits to economic development and societal progress [1,2]. Figure 1 shows examples of smart devices developed for use in our daily lives including smart sensors, smart homes, smart monitoring devices, smart portable electronics, smart vehicles, smart machines, smart IoT devices, and smart robotics. These devices possess characteristics suitable for a wide range of practical applications such as smart grids for power management [3], smart devices for monitoring and detecting problems associated with human health [4], and in smart manufacturing systems to enhance prediction ability [5]. SDs have been developed for smart transportation systems that interconnect with each other [6] and have been applied in SD-based triboelectric nanogenerators for biomedical, logistic, healthcare, and wearable applications [7,8]. Emerging technologies have a critical influence on human living, improving our material and spiritual lives in applications such as energy harvesting [9], biotechnology [10], intelligent robotics [11], functional and advanced material development [12], and nanotechnology for cancer therapy [13]. Emerging technology has played an important role in the development of modern society such as in the building of smart city models and in information and communication [14]; the Internet of Things (IoT); machine learning, computing, and big data in Industry 4.0 [15]; and in additive manufacturing as an emerging technology for many applications such as aerospace and nanoscale fabrication [16]. Figure 2 demonstrates the successful emerging technologies that have been developed for practical applications including triboelectric nanogenerators, geospatial technology, artificial intelligence with wearable applications, big data, robotics, smart applications, blockchain, additive manufacturing, advanced materials, Internet of Things (IoT), and nanotechnology. However, smart devices and emerging technologies need energy to drive their applications. Energy has received significant attention because it is indispensable for all of our daily activities, including machines, devices, and equipment. Traditional energy comes from fossil fuel resources such as oil and coal, but the exploitation and processing of these fuels cause destruction to our living environment [17]. Renewable energy (RE) has recently emerged as an energy source that can replace traditional energy [18]. Renewable energy exhibits the desirable features of being green, emitting no gases, and being environmentally friendly and can be obtained from solar energy, hydropower, wind energy, triboelectric nanogenerators, and ocean energy [19]. Renewable energy usage has increased considerably to meet global energy consumption needs. The renewable energy generation capacity (REEGC) reached 3864.52 gigawatts (GW) in 2023, higher than that of 3391.35 GW in 2022, corresponding to an increase of 13.98 percent, and it is expected to reach approximately 11,174 GW by 2030 (by following the International Renewable Energy Agency (IRENA)’s 1.5 °C pathway with preventing the global temperature from increasing by more than 1.5 °C by 2030), corresponding to an increase of 229.52 percent in comparison to the value recorded in 2022 [20]. Table 1 lists detailed renewable electricity generation capacities by region from 2022 to 2024. The table illustrates the remarkable energy transition from fossil fuels to renewable energy all over the world; in particular, outstanding records from Asia show an REEGC of 1631.02 GW in 2022, 1960.96 GW in 2023, and 2382.47 GW in 2024, corresponding to increases of 25.33%, 50.68%, and 83.07% compared with 2020. The REEGC values from Europe are also impressive at 705.14 GW in 2022, 778.51 GW in 2023, and 848.63 GW in 2024, corresponding increases of 16.35%, 28.46%, and 40.03% compared with 2020. North America area has a significant renewable energy transition, with REEGC values of 492.47 GW in 2022, 527.11 GW in 2023, and 573.01 GW in 2024, corresponding increases of 15.95%, 24.10%, and 34.91% compared with 2020. Figure 3 shows the percentages of REEGC achievements from 2020 to 2024 in comparison to the goals set by the 2030 and 2050 Scenario for lowering global temperature of 1.5 °C, respectively. The figure shows that the REEGC increases year by year from 2020 to 2024, but is still low, with the maximum percentage of 39.81% being reached in 2024 in comparison to the goal of Scenario 2050 and the minimum percentage of 8.47% being reached in 2020 in comparison to the goal of Scenario 2030, respectively [21]. The evidence reveals that the goal of the non-stop development of energy harvesting to meet IRENA’s 1.5 °C scenario is on track. There are some new energy harvesting production techniques that can help meet our daily consumption requirements, such as solar energy [22], wind energy [23], wave energy [24], hydropower [25], geothermal energy [26], ocean energy [27], and bioenergy [28]. The triboelectric nanogenerator (TENG) is an emerging technology that generates energy for a range of electrical devices such as microfluidic devices [29], sensors, smart devices, portable phones, health monitoring applications, and wearable devices [30]. TENGs are a type of modern energy conversion technology that is used to harvest wasted mechanical energy and transform it into electrical energy [31]. TENGs possess outstanding characteristics that are useful for smart devices and emerging technologies such as blue energy for monitoring marine environments [32], green energy for portable electronics [33], the ability to drive MEMS systems [34], renewable energy [35], the capacity to integrate into IoT systems [36], low cost, flexibility [37], light weight, and self-powered energy [38]. TENGs are also highly effective when applied in modern technologies such as in sensors for soft robotics and digital twin functions [39], force sensing with high sensitivity [40], and integration with machine learning for IoT applications [41]. TENGs produce electric power through the coupling of triboelectric materials, with one material donating free electric particles and another receiving the free electrical particles. Following this, charge transfer takes place between the two tribo-materials in the working cycle by coming to contact with each other and separating from each other to produce an electric flow running via external resistance [42]. TENGs possess many good characteristics, including biodegradability [43], being constructed from organic materials [44], sustainable power [45], green energy [46], multifunctionality [47], high energy conversion yield [48], and self-powering sources [49]. TENGs are special electric generators because they are environmentally friendly and do not emit gases such as carbon dioxide and methane [50]. Sustainable energy production can be guaranteed using TENGs; the energy lost during the transmission process is reduced, and the costs of energy consumption can be guaranteed at a reasonable and affordable price [51]. TENGs have recently received considerable attention from researchers and engineers for development in practical applications, including as a self-powered source [52] and sustainable energy resource [53], in renewable energy and green energy applications [54], and in Internet of Things utilizations [55]. TENGs have been successfully developed for diverse applications such as in biomedical sensors [56], ocean monitoring systems [57], smart agriculture development [58], in industrial fields [59], and in fluid dynamic sensors [60]. TENGs have been successfully employed in smart devices and emerging technologies such as in IoT models [61], monitoring the operating of fire alarm systems [62], in machine fault detection frameworks [63], in smart farming strategies [64], and in the development of degradable TENGs in information transfer systems [65]. TENGs can also be effective in practical applications such as human–machine interface solutions [66], self-powered robotics [67], smart healthcare purposes [68], self-powered sensors [69], wireless weather sensing networks [70], voice warning devices [71], wearable electronic systems, self-powered clothing [72], pressure sensors [73], and multifunctional applications [74]. Some TENGs are devised using nano/micro-structured materials for critical applications; examples include a TENG with a power of 10 mW constructed from PVDF nanofiber for water splitting [75], a TENG made of Au/PTFE with micro/nano structures showing a voltage of 0.12 V and current of 40 µA for food intake adjustment [76], and a TENG constructed from microneedle PDMS/Al for charging and lighting applications [77]. TENG has some limitations related to their output performance such as low power [78] and fluctuating output in alternating weather conditions [79]. Solutions include the development of new techniques and materials [80], enhancing the output performance at the early stage of design and optimizing processes for TENGs [81], stabilizing working conditions by introducing TENGs into multi-harvesting energy resources [82], and managing the output performance of the TENG via power management systems [83]. This paper reviews recently developed triboelectric generators and their useful applications for smart devices and emerging technologies. It is hoped that triboelectric nanogenerator technology will be expanded for further practical applications in smart devices and emerging technologies in the near future.

## 2. Operating Principles of Triboelectric Nanogenerators and Hybrid Renewable Energy Systems

Through the triboelectric effect, TENGs convert mechanical energy into electricity using coupled triboelectric materials under contacting–separating cycles. TENGs have four working mechanism modes for producing electrical energy: contact–separation mode, sliding mode, free-standing mode, and single electrode mode. TENGs have recently emerged as an advanced energy harvesting technology that is modern, green, renewable, flexible, and endurable. High-performance TENGs, denoted as advanced triboelectric nanogenerators, have potential for use in many practical applications such as self-powered sources and portable electric devices, self-powered sensors, power grid penetration, manufacturing process monitoring, quality control, and medical and healthcare applications that meet the criteria of smart devices and emerging technologies. Advanced TENGs are highly efficient energy harvesters that can convert many types of wasted mechanical energies into the electric energy used in a range of practical applications such as smart devices, portable electric devices, smart sensors, smart manufacturing systems, and automatic actuators. Advanced TENGs feature many valuable characteristics of an energy source for smart devices and emerging technologies such as cost effectiveness, sustainable and renewable energy, green energy, light weight, flexibility, self-powered energy, and portable energy sources. Advanced TENGs are based on the triboelectric effect to harvest wasted mechanical energy and transform it into electricity [84]. Advanced TENGs feature outstanding characteristics of advanced sustainability [85], advanced flexibility [86], and blue energy [87]. TENGs generate electricity based on the triboelectric effect that occurs during the repeated contact and separation of two triboelectric materials. According to the triboelectric principle, one tribo-material provides electrical charge particles, while the other receives electrical charge particles [88]. Abundant tribo-materials are available in our environment that can be used for making triboelectric nanogenerators such as eco-materials, wood, metal, and polymers. The four basic operation modes of TENGs and hybrid renewable energy systems are described below.

### 2.1. TENG Based on Contact–Separation Mode (CS-TENG)

**Figure 4:**
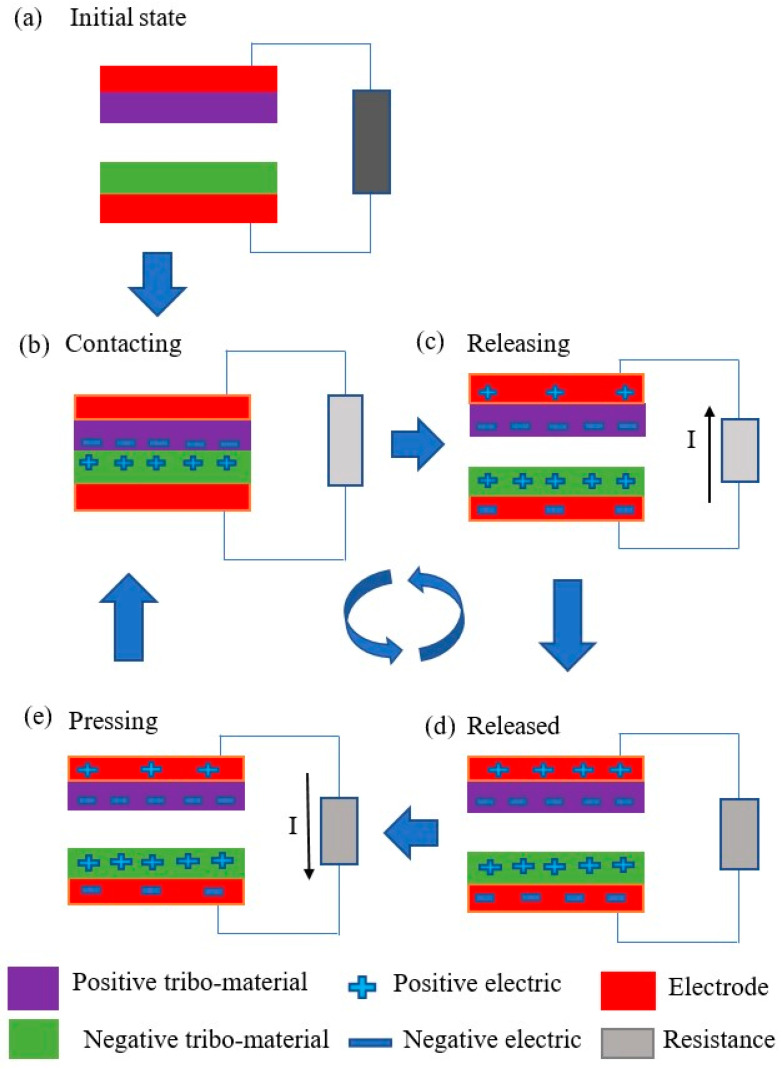
The working mechanism of the TENG, including four steps of cyclic contact and separation to create electricity; (**a**) The initial position with no charges; (**b**) The two tribo-surfaces contacting with each other to generate electricity; (**c**) The separating state causing unbalanced potentials between the two triboelectric materials; (**d**) The released position of the two triboelectric materials with a balanced state; (**e**) The subsequent pushing state causing unbalanced potentials between the two triboelectric materials.

Figure 4 illustrates the working mechanism of the TENG during contact–separation mode. It includes four steps of cyclic contact and separation to create electricity. Figure 4a illustrates the initial position when no charges occur. Figure 4b illustrates the two tribo-surfaces that move into contact with each other to generate electricity. Figure 4c illustrates the separating state, which produces unbalanced potentials between the two triboelectric materials. The electric flow runs via the external load. Figure 4d illustrates the released position of the two triboelectric materials with a balanced state. Figure 4e illustrates the subsequent pushing state that generates unbalanced potentials between the two triboelectric materials. The current runs via the external load. The triboelectric nanogenerator creates electricity represented by open-circuit voltage (*V_OC_*), as demonstrated by Formula (1) [89]:

(1) $$V_{OC}=-\frac{\sigma d}{\varepsilon_{0}}$$

where $V_{OC}$ is the open-circuit voltage; σ is the triboelectric charge density; d is the distance between the two contact surfaces; and $\varepsilon_{0}$ is the vacuum permittivity.

### 2.2. TENG Based on Sliding Mode (Sl-TENG)

**Figure 5:**
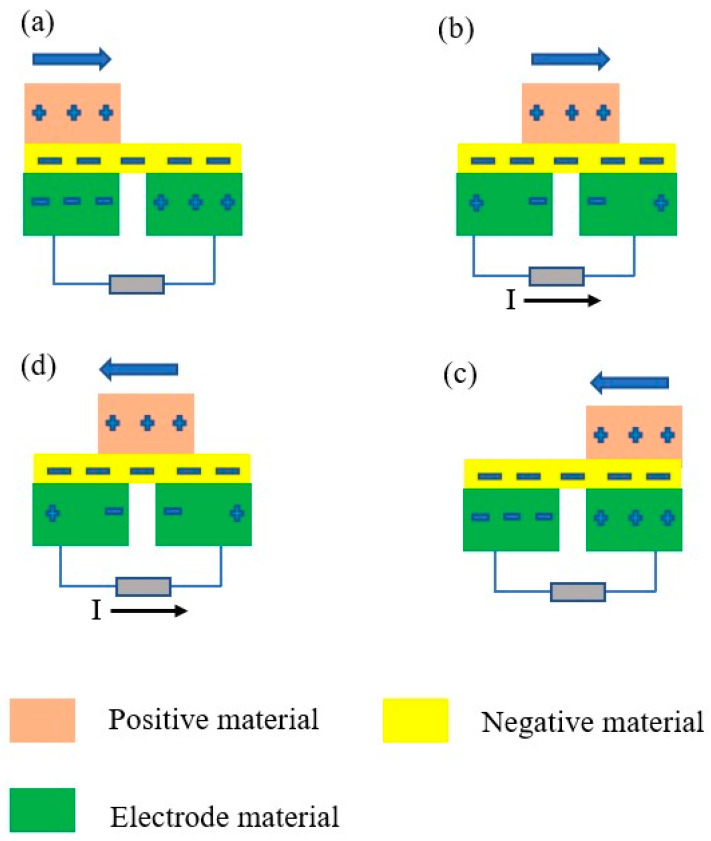
The working mechanism of the sliding-mode triboelectric nanogenerator to produce electricity; (**a**) The initial stage with no charges; (**b**) The two tribo-surfaces that slide into contact with each other to produce electricity; (**c**) The delay state, which recovers the balanced potentials between the two triboelectric materials; (**d**) The two tribo-surfaces that slide into contact again with each other to produce electricity.

In sliding mode, the triboelectric nanogenerator undergoes four stages of sliding-in-contact between two triboelectric materials—positive and negative—to produce electricity. Figure 5 shows the sliding mode mechanism of the triboelectric nanogenerator. Figure 5a illustrates the initial stage where no charges occur. Figure 5b illustrates the two tribo-surfaces that slide into contact with each other to produce electricity. The electric flow runs via the external load. Figure 5c shows the delay state, which recovers the balanced potentials between the two triboelectric materials. Figure 5d illustrates the two tribo-surfaces that slide into contact again with each other to produce electricity. The current runs via the external load [90]. The open circuit voltage of the sliding TENG can be expressed by Equations (2)–(4) [91]:

(2) $$Voc=\Phi_{1}-\Phi_{2}$$

where *V_OC_* is the open circuit voltage of the sliding TENG; *Φ*_1_ denotes the potential of the triboelectric material 1; and *Φ*_2_ represents the potential of triboelectric material 2.

(3) $$\Phi_{1}=\frac{\sigma_{T}}{\pi \varepsilon_{1}}\left[\int_{d_{1}}^{\infty }f\left(x\left(t\right),z\right)dz\right]$$

(4) $$\Phi_{2}=\frac{\sigma_{T}}{\pi \varepsilon_{2}}\left[\int_{d_{2}}^{\infty }f\left(x\left(t\right),z\right)dz\right]$$

$\varepsilon_{1}$ is the dielectric constant of layer 1; $\varepsilon_{2}$ represents the dielectric constant of the layer 2; *x*(*t*) is velocity of the movement of the triboelectric layer; and $\sigma_{T}$ denotes the charge density of triboelectric surface.

### 2.3. TENG Based on Free-Standing Mode (FS-TENG)

**Figure 6:**
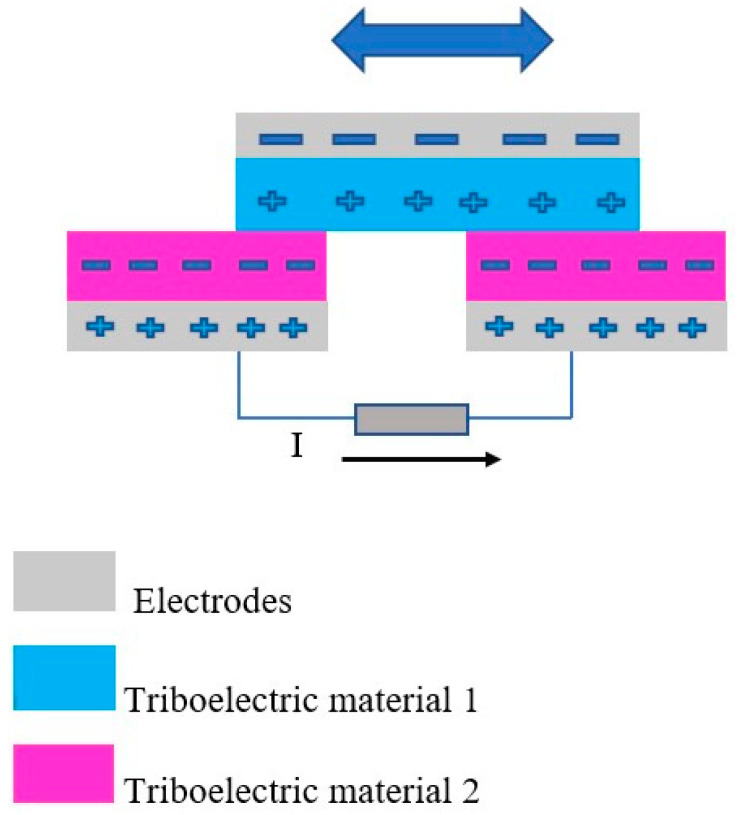
The working principle of the free-standing-mode triboelectric nanogenerator when generating an electric current.

In free-standing mode, the triboelectric nanogenerator produces a current via an external load by acting of one dielectric layer on the two dielectric surfaces. The dielectric layer activates electrical particles moving between the two electrodes, resulting in a current running via an external load. Figure 6 shows the working principle of the free-standing-mode triboelectric nanogenerator when generating an electric current via an external load [92]. The basic governing equation for the output performance of the free-standing TENG can be briefly expressed by Equation (5) [93]:

(5) $$R\frac{dQ}{dt}=V=-\frac{1}{C}\times Q+V_{OC}$$

where *R* is the resistance; *Q* denotes the charge on the tribo-material; *V* is the output voltage; *V_OC_* is the open circuit voltage; and *C* is the capacitance.

### 2.4. TENG Based on Single Electrode Mode (SI-TENG)

**Figure 7:**
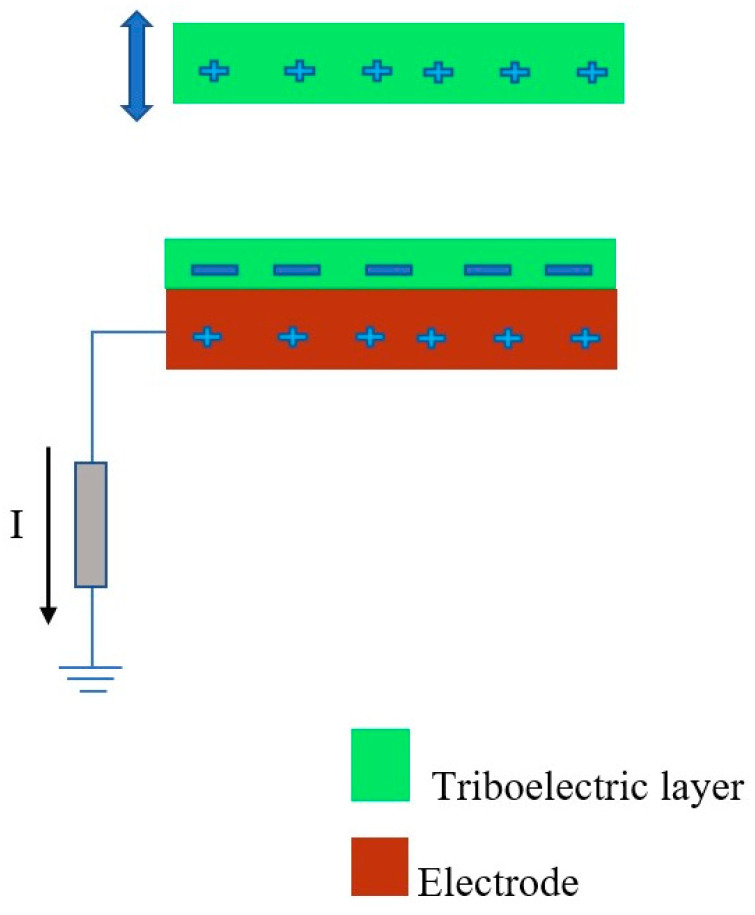
The working mechanism of the single-electrode-mode triboelectric nanogenerator when harvesting electricity.

A single-electrode-mode TENG (S-TENG) consists of triboelectric layers and one electrode. The S-TENG is combined with another electrode to direct the electric current moving out of the S-TENG during the production of electricity, as shown in Figure 7 [94]. The electrical characteristics can be expressed by the following governing equation (Equation (6)) [95]:

(6) $$V=-\frac{1}{C}\times Q+V_{OC}$$

where *R* is the resistance; *Q* denotes the short circuit charge of the tribo-material; *V* is the output voltage; *V_OC_* is the open circuit voltage; and *C* is the capacitance.

### 2.5. TENG for Hybrid Renewable Energy Systems (HRESs)

**Figure 8:**
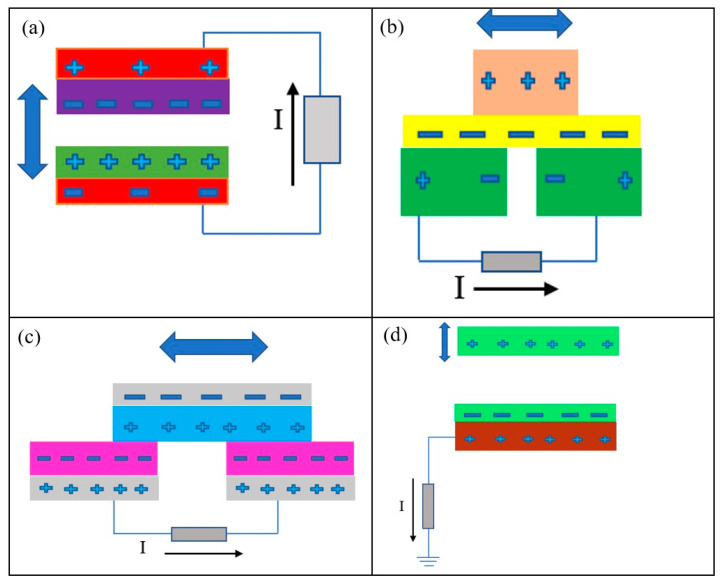
The four working principle modes of the advanced TENG: (**a**) contact–separation mode; (**b**) sliding mode; (**c**) free-standing mode; (**d**) single electrode mode.

One of the biggest roles of the TENG is that it can be used in hybrid energy systems and power grids to boost the availability of energy for daily living [96]. Advanced TENGs can produce electricity using the four working principle modes shown in Figure 8: contact–separation mode, as shown in Figure 8a; sliding mode, as shown in Figure 8b; free-standing mode, as shown in Figure 8c; and single electrode mode, as shown in Figure 8d, respectively [97].

**Figure 9:**
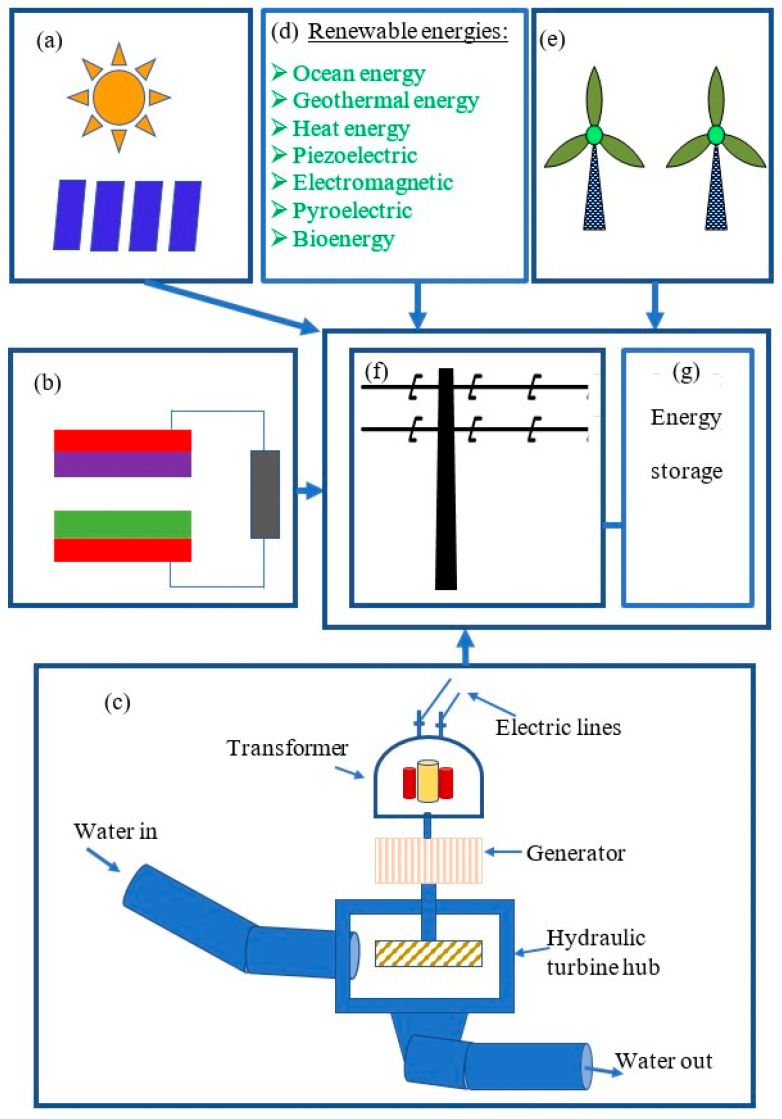
The proposed hybrid renewable energy system. (**a**) Solar energy; (**b**) triboelectric nanogenerator; (**c**) hydropower; (**d**) renewable energy harvesters of ocean energy, geothermal energy, heat energy, piezoelectric generators, electromagnetic generators, pyroelectric generators, and bioenergy; (**e**) wind power; (**f**) power grid; (**g**) energy storage.

A hybrid renewable energy system (HRES) is an energy system that consists of two or more energy sources such as solar energy, wind power, ocean energy, geothermal energy, heat energy, piezoelectric generators, water wave energy, triboelectric nanogenerators, electromagnetic generators, pyroelectric generators, and bioenergy [98]. HRESs can support energy for a limited area and be integrated into power systems such as power grids and microgrids to transfer energy to remote areas and areas that lack electrical facilities. Hybrid energy systems obtain more benefits by combining with renewable energy sources to reduce carbon dioxide [99], optimizing the output performance of the energy system [100], and reducing the impact of external exciting conditions on the output performance of single renewable energy systems such as region featuring, weather conditions [101], and fluctuating output performance [102]. TENGs have been proven to make a positive contribution to energy development all over the world, and are deeply integrated with energy systems, for example, with a piezoelectric nanogenerator (PENG) in a hybrid energy system (HES) developed to improve charge density and electrical yield for flexible electronics [103], and with an electromagnetic generator (EMG) in an HES to produce electricity. Figure 9 illustrates a proposal hybrid energy system involving a TENG to produce electric energy. The figure shows that hybrid renewable energy conversion methods and components that can be integrated into the power grid and energy storage unit include solar energy, as shown in Figure 9a; triboelectric nanogenerators, as shown in Figure 9b; hydropower, as shown in Figure 9c; renewable energy harvesters of ocean energy, geothermal energy, heat energy, piezoelectric generators, electromagnetic generators, pyroelectric generators, and bioenergy, as shown in Figure 9d; wind power, as shown in Figure 9e; power grids, as shown in Figure 9f; and energy storage, as shown in Figure 9g. Through integration into a hybrid energy system, a single renewable energy power source like a TENG can bring advantages to the power grid, such as providing stable self-powered energy, balancing output performance, and converting wasted energy from the surrounding environment into useful energy.

**Figure 10:**
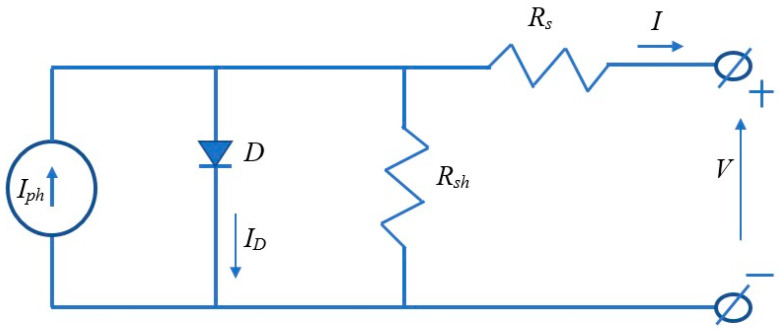
The equivalent circuit that converts solar energy into electricity using a single solar cell.

Solar energy is normally converted into electricity using a solar electricity energy converter. One example is a solar photovoltaic system, which is used to change solar energy to electrical energy under the photovoltaic effect of solar cells fabricated from semiconductors with junctions of p-n. Figure 10 shows the equivalent circuit that converts solar energy into electricity using a single solar cell. Solar cells produce power with an output current and output voltage as illustrated in Equation (7) [104]:

(7) $$I=I_{ph}-I_{0}\left\{exp\left[\frac{q\left(V+IR_{s}\right)}{AkT}\right]-1\right\}-\left(\frac{V+IR_{s}}{R_{sh}}\right)$$

where *I* is the output current of the solar cell; *I_ph_* denotes the photovoltaic current of the solar cell; *I*_0_ represents the saturation current; *q* denotes the electric charge of the PV cell; *V* is the output voltage of the solar cell; *R_s_* presents the shunt resistance; *A* is the fitting factor; *k* is the Boltzmann constant; and *T* denotes the absolute temperature.

Piezoelectric generators change mechanical energy into electricity based on the piezoelectric effect, which can be expressed by Equation (8) [105]:

(8) D=ε·E

where *D* denotes the charge density displacement; *ε* denotes the permittivity of the piezo-materials; and *E* denotes the strength of the electrical field.

The piezoelectric harvester produces a short circuit current, as in expression (9):

(9) $$I=\frac{dD}{dt}$$

where *I* is the short circuit current and *t* is time.

Hydropower is one type of renewable energy source that produces electrical energy by directing the water flow moving through a turbine. A hydropower plant generates potential power as per Equation (10) [106]:

(10) p=ρ·g·Q·H·η

where *p* represents the potential power of the energy system (W); *ρ* denotes the density of the water flow (kg/m^3^); *η* denotes the overall efficiency; *g* is the gravitational acceleration (m/s^2^); *Q* denotes the water volume flow rate (m^3^/s); and *H* represents the available head (m).

The economic efficiency of a hybrid energy system can be estimated using expression (11) [107]:

(11) $$F(o)=\sum_{i}\left(\sum_{j}C_{j}-\sum_{l}I_{l}\right)$$

where *F*(*o*) is the objective function of the hybrid energy system that is normally expectant to minimize; *C_j_* represents the electrical cost of the *j* renewable energy source; and *I_l_* denotes the income value.

The objective function is best when the energy cost is minimized and the income is maximized.

In brief, TENGs show good characteristics for further development in the field of energy harvesting. Many research groups are focusing on developing TENGs for special applications and integrating them into hybrid systems, such as developing advanced device mechanisms involving liquid-metal-based TENGs to improve output performance [108], developing a hybrid TENG-based triboelectric nanogenerator and piezoelectric nanogenerator to improve energy harvesting efficiency [109], and introducing a hybrid TENG harvester based on a triboelectric nanogenerator and electromagnetic generator to enhance the output performance of the energy harvester system for agriculture applications [110]. Other special applications have been introduced by research groups, such as developing a hybrid self-powered sensor-based triboelectric nanogenerator and electromagnetic generator [111], improving the output performance of a flexible TENG using nanograting for PDMS layers [112], and constructing flexible TENG devices with membranes crosslinked with silver layers [113]. Due to its outstanding features, the TENG is emerging as a new technological trend in modern energy and renewable energy in all practical applications from energy production to material technology and manufacturing related to smart technologies, smart devices, and emerging technologies.

## 3. Smart Device and Emerging Technology Applications

### 3.1. Smart Devices (SDs)

**Figure 11:**
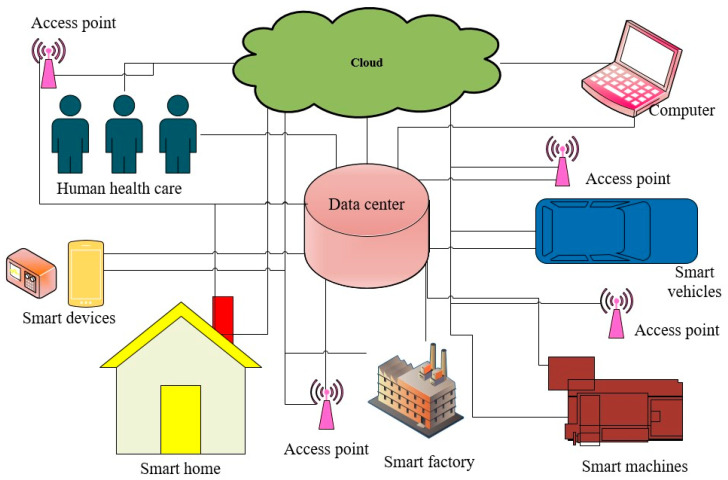
Smart devices integrated into an IoT system including smart homes, smart factories, smart machines, smart vehicles, and smart healthcare devices.

Smart devices are electronic devices that possess the special characteristics of context-awareness, the ability to interconnect with other equipment in the operating system, and autonomous computation. Figure 11 shows smart devices integrated into IoT systems such as smart homes, smart factories, smart machines, smart vehicles, and smart healthcare devices [114]. Smart devices are low in cost, flexible, smart, stable, and highly accurate and can be used in real time [115]. Smart devices are now utilized in many fields of practical applications such as in additive manufacturing [116], smart grids [117], remote laboratory control [118], personal protection devices for women [119], smart homes to make people more comfortable [120], and in the management of human diabetes [121]. In the interconnecting environment, smart devices can connect to other electronic components through IoT gadgets, share electronic data, process autonomous duties related to signals and data, communicate effectively with electronic cells, and improve the quality of system performance. Some practical applications of TENGs have attracted research groups such as using smart devices to improve the positioning precision of global navigation satellite systems [122], monitoring heartbeats through acoustic signals [123], introducing wearable aerogel into smart devices for controlling the temperature [124], integrating smart devices into sensor networks for monitoring the ecosystem via the IoT [125], and measuring noise exposure with high accuracy [126]. The proliferation of application-oriented smart devices has expanded the smart environment in which smart devices are proactive in communicating and processing data to perform smart tasks in a smart platform with integrated smart devices, internet connection, digital computing, and protocol interfaces.

### 3.2. Emerging Technologies (ETs)

**Figure 12:**
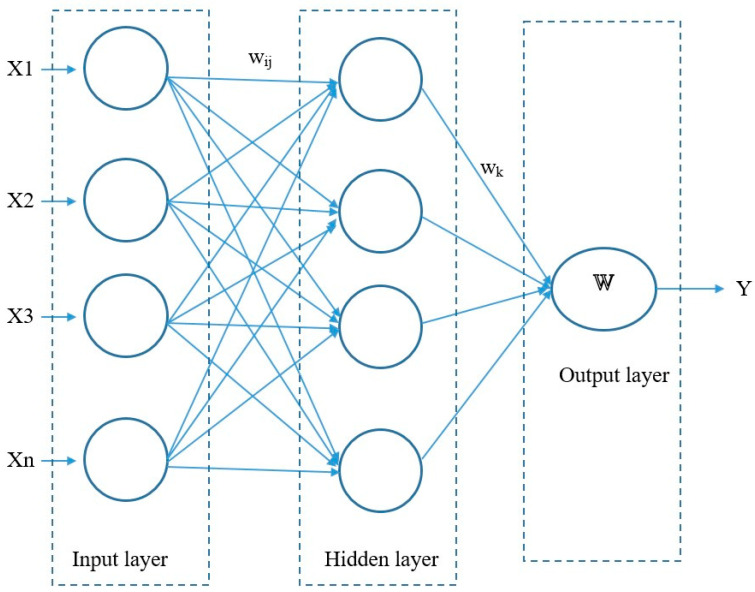
Neural network model for analyzing and processing data for emerging technologies.

Emerging technologies possess features that contribute the emergence of critical new technology and have a huge innovative impact on modern society in all aspects of daily living [127]. Emerging technology can improve aspects such as the economy, technology, education, and society [128]. Many emerging technologies have been utilized successfully in areas such as big data [129], geospatial technology, artificial intelligence [130], robotics [131], the Internet of Things (IoT) [132], smart applications, blockchain [133], nanotechnology [134], and additive manufacturing [135]. Machine learning models are effective methods used to analyze and process a large amount of data for emerging technologies [136] with many benefits including rapid data processing, cost effectiveness, shortened time, and the ability to predict results [137]. Machine learning models include supervised and unsupervised learning tools [138] developed for many applications such as analyzing data for emerging technologies (data analysis, geoinformation, machine learning, and artificial intelligence methods) related to transportation in smart cities [139]. Some successful introductions into practical applications include the development of a neural network-based machine learning model for semiconductor technology with the ability to capture the electrical responses of a transistor [140]; integrating the emerging technologies of unmanned aerial vehicles, non-orthogonal multiple access, and intelligent reflection for a sixth-generation communication network [141]; and using machine learning to analyze the power flow of a PV power network [142]. Figure 12 shows a neural network model used to analyze and process the data for emerging technologies related to solving technical problems including minimizing defects, optimizing processing parameters, and improving service quality. The neural network model basically utilizes a mathematical algorithm to imitate the biological behaviors and neurobiology of living objects such as the human neural network. The model includes three basic parts with an input layer, hidden layer, and output layer to process the data based on machine learning. The input layer supports technical processing parameter values, such as the x vector, of hidden layers. The hidden layer transfers its result to the output layer using a mathematical function. The output layer finalizes this by processing the signal from the hidden layer to meet the goal of the designer [143]. The model is efficient for predicting goal of technical problems [144]. The general mathematic algorithm can be demonstrated as follows (Formula (12)):

(12) $$y=f\left(W\chi \right)=\sigma \left[\sum_{j=1}^{n}\alpha_{jk}\sigma (\sum_{i=1}^{m}x_{i}w_{ij}+a_{j}^{h})+a_{k}^{0}\right]$$

where *x_i_* refers to variables that describe the input parameters related to the technical system; *w_k_* denotes the weighted features related to the variable neurons; *w_ij_* represents the weight between the *i^th^* input node and the *j^th^* hidden unit; $a_{j}^{h}$ represents the bias of the *j^th^* hidden node; and $a_{k}^{0}$ represents the bias of the *k^th^* output node.

Smart devices and emerging technology (SDET) are effective and successful in real applications such as in ultrasound, non-thermal plasma applications, and ultraviolet light treatment techniques for enhancing the germination of seeds in agriculture [145], as well as using information and communication technologies (ICT) for building information modeling (BIM) with emerging technologies such as sensor networks, sematic webs, and cloud computing to enhance performance [146]. Some SDETs have been developed and effectively utilized for practical applications such as pulsed electric field, high-pressure processing, pulsed light, ultrasound, irradiation, cold atmosphere plasma, and oscillating magnetic field applications to improve the quality of food processing [147], and for drug safety such as nano-technology, vitro phenotyping with machine learning and imaging, bioinformatics, and omics techniques [148]. Some vital SDETs have received attention from researchers such as the management of Industry 4.0 applications including the Internet of Things, artificial intelligence, blockchain, 3D printing, virtual and augmented reality, cloud computing, big data, biometrics, biotechnology, nanotechnology, and smart factories [149], and using emerging technologies for driving digital manufacturing systems such as digital twin and optimization techniques, cyber–physical systems [150], and bio-robotic developments in biology, control science, physics, chemistry, and material science [151].

### 3.3. Advanced TENG for Smart Devices and Emerging Technologies

**Figure 13:**
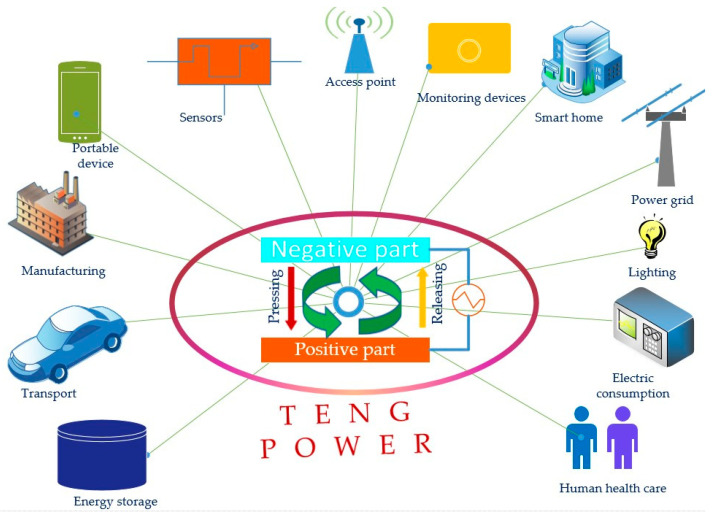
The proposed model that uses an advanced TENG for smart devices and emerging technologies such as energy storage, transportation, manufacturing systems, portable devices, smart sensors, self-powered sensors, access points, monitoring devices, smart homes, power grids, lighting applications, electric consumption devices, human healthcare, and smart agriculture production.

With its outstanding characteristics, the TENG is a suitable candidate for use in smart devices and emerging technologies such as modern energy, renewable energy, portable energy, sustainable energy, and self-powered sources. Smart devices and smart manufacturing systems involve a range of advanced technologies such as digitalization technology, process automation, prediction technology, data storage, agent technology, cloud computing technology, and AI technology [152]. Smart sensors are vitally important in the control and management of these systems. TENGs provide many ways of powering the sensors in smart manufacturing networks, such as providing supporting power to drive Internet of Things devices in smart manufacturing [153], driving the fluid flow for a smart factory [154], human–machine interface applications [155], and introducing sustainable tribo-materials into smart manufacturing systems [156]. Emerging technologies possess many outstanding features: they can be used for high yield benefit [157], innovation development [158], and application potential [159]; are modern in character [160] and can be used in modern and new energy [161]; and can be used for social improvement, digital-related technologies, sustainability-related technologies, the Internet of Things, artificial intelligence, big data, computing technology, sensing penetration, machine learning, and predicting techniques, attracting much attention from researchers [162,163]. Emerging technologies also include new and modern technologies such as digital blockchain technology [164], plasma technology for energy storage [165], solar reformation for recycling waste and for the chemical industry [166], intelligent robotics for manufacturing, healthcare, and logistics [167], 5G network communication technology for UAVs [168], IoT technology for smart construction [169], Internet of Things technology for medical devices [170], nanoparticles and biosynthesis for sustainable agriculture [171], and tumoroid technology for lung cancer treatment [172]. Some TENGs show potential ability in the development of SDET such as in therapeutics [173], TENGs for drug delivery [174], TENGs with ultra-durability for transforming wasted mechanical energy into electricity [175], TENGs for smart fabrics and emerging wearable technologies [176], TENGs for smart medical sensors to monitor human health [177], integrating TENGs and electromagnetic generators (EMGs) for smart agriculture models [178], and TENGs for self-powered sensors integrated into machine learning techniques [179]. Figure 13 shows the proposed model that uses an advanced TENG for smart devices and emerging technologies such as energy storage, transportation, manufacturing systems, portable devices, smart sensors, self-powered sensors, access points, monitoring devices, smart homes, power grids, lighting applications, electric consumption devices, human healthcare, and smart agriculture production [180]. High-performance TENGs, denoted as advanced triboelectric nanogenerators, have potential for use in many practical applications such as self-powered sources, self-powered portable electric devices, self-powered sensors, power grid penetration, monitoring manufacturing processes, controlling quality, and medical and healthcare applications that can meet the requirements for smart devices and emerging technologies.

TENG has the novel characteristics of being self-powered, flexible, and exhibiting stability during the working process, all of which are important in smart devices and emerging technologies. Triboelectric nanogenerators have been developed and deployed for many applications related to SDET, such as supplying power to monitor alarms and geological disaster systems [181], applying self-powered sensors for a sport sensing network in a smart sports equipment facility [182], fabricating TENGs for self-powered gas sensing application [183], using TENGs with artificial intelligence (AI) [184], and building a TENG to convert ocean energy into electricity [185]. Other potential goals that have been achieved by researchers include developing a TENG for digital twin applications for capturing movement behavior [186], constructing a TENG as a self-powered power source for sensing systems [187], fabricating a TENG for use in microbial disinfection [188], using a TENG as a chemical sensor [189], combining a TENG with an Internet of Things (IoT) system [190], using a TENG in wearable devices [191], utilizing a TENG in medical health monitoring [192], and using a TENG in self-powered systems [193]. Table 2 illustrates the yield and applications of TENGs that have benefited smart devices and emerging technology in practical applications. The table shows that TENGs have been utilized in many practical applications from electrical production, lighting, charging, smart sensors, IoT technology, electronic devices, smart devices, bio-implants, and bio-devices.

### 3.4. TENG and Potential Applications

It is clear that TENG technology can be used to harvest abundant wasted mechanical energy. There are many potential applications for TENGs, such as harvesting respiration energy for self-powered biomedical devices [194], constructing a TENG from graphene oxide for self-powered smart water filter machines and potential multipurpose smart sensing applications [195], self-powered sensors to observe the ocean current at deep water levels [196], biomimetic vehicles with potential ability for digital twin control [186], controlling robot and artificial intelligence applications [197], and producing a TENG for biometric techniques with potential applications in portable electronics, machine learning, and handwriting sensors [198]. TENGs can be feasibly integrated into a hybrid system to produce energy and can be introduced into further applications such as a hybrid TENG and graphene material system for potential self-powered electrocatalytic techniques in wastewater purification applications [199], using a nanocomposite film of polyvinyl alcohol and silver nanoparticles to harvest wearable energy for sensor devices [200], and using silver nanoparticles and crosslinked PDMS for smart insoles and alarm systems [201]. TENG can be combined with other energy harvesters in a hybrid energy system for better energy harvesting efficiency [202] and productivity [203], and for filling gaps [204]. They can also be used to improve applications: a hybrid TENG and electromagnetic energy system for smart agriculture and smart sensing devices [205], a hybrid TENG and piezoelectric nanogenerator system for wearable wireless equipment [206], a hybrid energy system (HES) constructed with piezoelectric and triboelectric nanogenerators for a self-powered monitoring device [207], and a hybrid TENG and photovoltaic energy alarm system [208]. TENGs show more potential applications than others, with outstanding comparative features including simple structure, flexible working, diverse materials, sustainability, self-powered energy, and high output performance [209]. Table 3 presents a comparison between the TENG and different energy conversion technologies such as EMG and PENG with the comparative criteria of material, working mechanism, energy production, advantages, disadvantages, and potential applications. The table shows the outstanding features of the TENG in comparison to other energy harvesting technologies such as electromagnetic generators and piezoelectric nanogenerators, with TENG exhibiting the highest voltage of 800 V. Most materials fit TENG requirements, and the TENG has flexible working mechanisms with four working modes, and a diversity of potential applications and advantages. In brief, the TENG is an emerging technology with outstanding characteristics that can be used alongside many current technologies such as material technology, information technology, biotechnology, nanotechnology, modern energy, renewable energy, smart technology, smart sensors, and hybrid technology systems for a vast number of practical applications, particularly for smart devices and smart technologies.

**Table 2:** Yield and applications of TENGs with advantages for smart devices and emerging technology in practical applications.

Materials	Surface Structure	Output Performance	Emerging Technology Applications	Refs.
Al/CP/BTO-PTFE/Cu	BTO nanoparticle	103 V, 3.6 µA, 32.4 µW/cm^2^	Charging, electronic device	[210]
Cu/PDMS-Ag/PDMS	Ag nanowire	*V_OC_* of 32 V, Isc of 0.7 µA, 9.36 mW/m^2^	Wearable strain sensor, self-powered clothing	[211]
PDMS/BSFO	BSFO nanomaterial	*V_OC_* of 152 V, Isc of 10.6 µA, 4.71 W/m^2^, 120 LEDs	Intelligent sensors	[212]
CNT/PEI Fabric	Carbon nanotube	3.2 W/m^2^	Small electronics, self-powered sensor	[213]
PTFE/fabric-Conductive fabric	Fabric	145 V (*V_OC_*), 3.25 µA (Isc), 343.19 mW/m^2^	Fire alarm network	[214]
PET/ITO/ZnO/PDMS-Al	ZnO nanorod	39.34 V (*V_OC_*), 82 µW	Pressure sensor, lighting	[215]
PVDF/Cu_2_Te-PVA/NaCl	Cu_2_Te leaf-structure	170 V, 32 µA, 1.62 W/m^2^	Wearable sensing, sustainable electronics	[216]
PDMS-ZnO	ZnO nanorod	39.34 V, 82.2 µW	Compression sensing	[215]
PHFC film	Nanofibers	330.6 µW/cm^2^	Sustainable agriculture	[217]
Diatomaceous earth/Carbon nanotubes/styrenebutadiene rubber	Nanotubes	6.26 mW/cm^3^	Sensor application	[218]
PDMS/Multiwall carbon nanotube	Carbon nanotube	110 V, 10 µA, 1 Wm^−2^	Internet of Things, human–machine interface (HMI) sensing	[219]
Electrochemical-TENG		575 V, 42 µA	Electromechanical applications	[220]
Polyaniline/Textile/PVC/Al	Textile	257.68 V, 5.36 µA	Wearable electronics, self-powered source	[221]
Ni_1_Co_2_Al-TENG	Textile	270 V, 18 µA	Self-charging system	[222]
Copper/PTFE/Copper	-	544 V, 61.16 µA, 33.27 mW	Portable electronics	[223]
Mg/PLA-Reed film/Mg	Cytoderm structure	0.176 V, 192 nA	Implanted medical device	[224]
Al/Nylon-TiO_2_ NPs/PV gel/ITO	TiO_2_ nanoparticles	121 V, 11.1 µA, 149 µW/cm^2^	Harvesting energy, temperature sensor	[225]
Skin-PVBVA@ MXene/Al	Eco-composite	252 V, 760 mW/m^2^	Sensing and harvesting energy	[226]

## 4. Advantages, Disadvantages, and Solutions

### 4.1. Advantages

**Figure 14:**
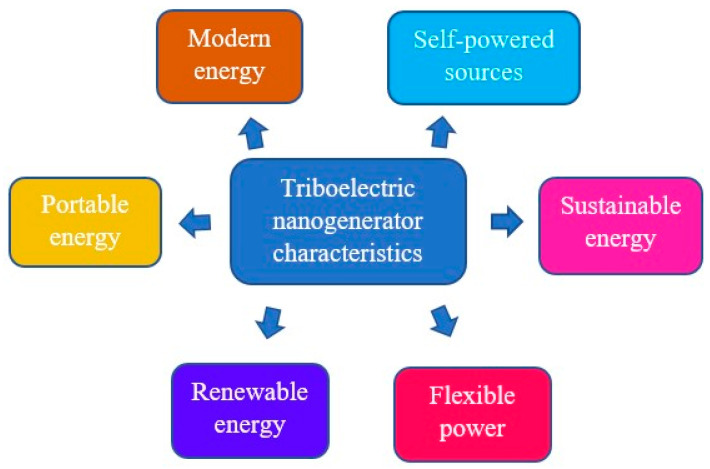
The advantages of TENGs in smart devices and emerging technologies.

Triboelectric nanogenerators have many advantages when introduced into smart devices and emerging technologies such as portable energy, modern energy, self-powered sources, renewable energy, flexible power, and sustainable energy, as shown in Figure 14. The specific advantages of TENGs are their self-powering ability, sustainable energy [331], low cost [332], flexible structure [333], blue energy [334], renewable energy, ability to be integrated into the power grid, and suitability for use in emerging technologies. Advanced triboelectric nanogenerators (ATENGs) possess the good characteristics of self-powered source, stability, and ability to convert many types of mechanical energy into electricity for smart devices, emerging technologies, and a variety of other applications [335]. TENGs have positive features such as stability, comfort, and sensitivity when introduced into self-powered sensor applications [336]. TENGs effectively convert biomechanical energy into electricity [337]. Although triboelectric nanogenerators can meet the requirements of smart devices and emerging technologies as mentioned above, there are still limitations. Based on these features, further research will boost the benefits for further applications and identify solutions to decrease the disadvantages for better servicing in smart devices and emerging technologies.

### 4.2. Disadvantages

The development of new energy sources like triboelectric nanogenerators is crucial to meet increasing energy consumption needs. However, rapid development inevitably comes with challenges such as technologies, materials for improving of triboelectric performance, TENG’s structure for increasing output performance, and sustainable working ability [338]. During development, some TENGs require expensive technologies such as the lithography technique to create tribo-patterns [339], additive manufacturing technology [340], hydrothermal technology to create TENG from molybdenum disulfide petals formed on electrospun polyacrylonitrile fiber [341], the spin coating technique and electrochemical anodization [342], and template etching to fabricate the microcolumn pattern of carbon nanotube (CNT)/polydimethylsiloxane composite [343]. New materials and tribosurface structures are normally developed using nano-structures and micro-structures to enhance output performance, but they are expensive and difficult to fabricate. Gold material is often used for the electrode in order to harvest electrical energy [344], the spin coating method is used to construct nanotube structures of nylon/carbon for the TENG [345], and nanopillar patterns are also used [346]. Some obstacles that have directly impacted TENG development relate to the triboelectric material used to improve electrical performance [347], the structure necessary to harvest the most energy from nature [348], the structure of the tribo-surface [349], the techniques and methods used to integrate the TENG into the power grid, the application of the TENG into new devices, the integration of energy into power grid, and output performance fluctuations [350]. Moreover, there are limitations associated with the technology used to store the energy obtained by the TENG for further applications [351], and it remains challenging to develop new TENGs with low-cost tribo-materials and shorter fabrication processes [352]. Some processes are required chemicals during the manufacturing steps [353]. Some other limitations relate to new technologies, mathematical methods, new functional materials for TENGs [354], and the application of smart devices and emerging technologies in critical fields with the ability to integrate TENG’s services in SDET [355].

### 4.3. Solutions

**Figure 15:**
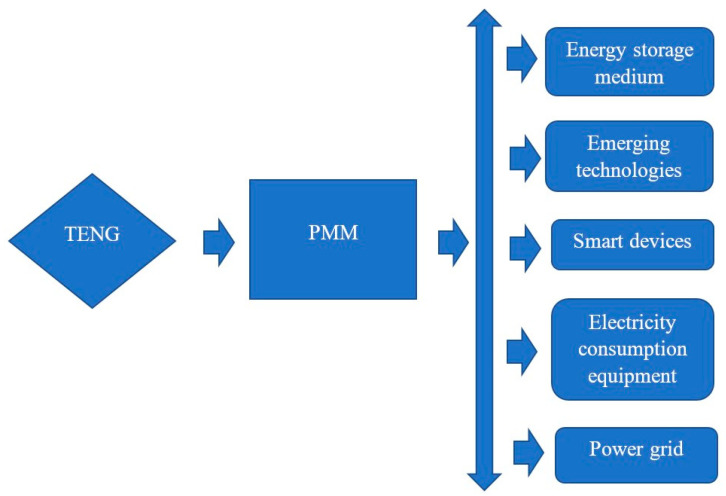
A power management module (PMM) to manage and boost the efficiency of the TENG in a power system for energy storage, powering emerging technologies and smart devices, powering electrical consumption equipment, and connecting to the power grid.

Solutions can be found to reduce the limitations of the TENG, including the use of new technologies, science, and materials to increase electric performance; using advanced structures, mathematical algorithms, and power management methods such as the chemical modification of polymer to improve output performance [356]; improving output performance by enhancing surface contact with special surface structures of micro-dome and micro-particle morphologies [357]; and introducing advanced-design TENGs into small power consumption equipment such as self-powered sensors for eco-smart cities [358]. Some solutions have shown promise, such as developing porous material to increase the output performance of the TENG [359], optimizing contact electrification via the surface modification of the TENG to enhance reliability and efficiency [360], developing advanced TENGs by applying chemical modification techniques [356], improving the output performance of the TENG with the electric displacement–internal field technique [361], developing a machine learning model for TENGs in sensing systems [362], and developing force-sensing TENGs using 3D printing technology [363]. Some critical solutions have been developed such as applying facet engineering to the metal–organic material Co-2-methylimidazole to enhance the charge density of the TENG [364], using plastic microneedle structures to enhance the surface contact of tribo-surfaces to increase output performance [365], developing hybrid emerging technology based on additive manufacturing for fabricating complex microelectromechanical systems [366], applying the simulation technique to simulate and optimize the performance of the energy harvesting system [367], using a power management kit and charge-boosting technique to meet the requirements of industrial and practical electric consumption applications [368], casting a dielectric layer of P25 TiO2 into the TENG to enhance its output performance [369], and applying new tribo-material to enhance output performance [370]. Other research groups have developed new technologies and methods to store energy from TENGs for long-term use by those in remote areas where access to energy is more difficult [371,372]. Figure 15 shows a power management module (PMM) to manage and boost the efficiency of the TENG in a power system for energy storage, powering emerging technologies and smart devices, powering electrical consumption equipment, and connecting to the power grid [373].

The emerging technology trend can be predicted by using forecasting methods such as the Gompertz model with the logistic rule in Equation (13) [374]:

(13) $$Y_{t}=Le^{{-ae}^{-bt}}$$

where

*Y_t_* is the Gompertz curve; *L* demonstrates the asymptotic coefficient of *Y_t_*; *a* is a location coefficient; and *b* demonstrates a shape coefficient.

(14) $$Y_{t}=\frac{L}{1+{ae}^{-bt}}$$

where

*Y_t_* is the Gompertz curve; *L* demonstrates the asymptotic coefficient of *Y_t_*; *a* is a location coefficient; and *b* demonstrates a shape coefficient.

These equations are effective tools in predicting the development trends of emerging technologies, such as carbon nanotube technology for the storage of hydro (H_2_), micro electro mechanical systems (MEMSs), nanolithography, organic LED display technology, assistive devices and smart devices in homes, hydro storage using metal hybrid technology, and hydro storage using sodium borohydride technology.

## 5. Conclusions

Smart devices and emerging technologies have become highly popular and have received significant attention for their ability to meet daily living needs, such as green technologies, reducing or replacing the human workforce, enhancing service performance, treating disease, monitoring healthcare, renewable energy, sustainable working, quality improvement, green energy, protecting the natural environment, sustainable energy, and non-gas emissions. Triboelectric nanogenerators (TENGs) have recently emerged as an advanced energy harvesting technology that is modern, green, renewable, and flexible, and provides an endurable energy resource for many practical applications such as self-power sources, self-powered portable electric devices, self-powered sensors, power grid penetration, manufacturing process monitoring, quality control, and medical and healthcare applications that utilize smart devices and emerging technologies. Advanced triboelectric nanogenerators are highly efficient energy harvesters that can convert numerous types of wasted mechanical energy into the electric energy used in a range of practical applications in our daily life, such as automatic actuators, smart sensors, portable electric devices, smart manufacturing systems, and smart devices. Advanced triboelectric nanogenerators possess many characteristics that are valuable in smart devices and emerging technologies, such as cost effectiveness, sustainable energy, renewable energy, green energy, light weight, flexibility, self-powered energy, and portable energy sources. This paper reviewed recently advanced triboelectric nanogenerators and their useful applications in smart devices and emerging technologies. The paper provides motivation for the development of the next emerging technologies for use in all areas of daily life. For smart devices and emerging technologies, TENGs can be applied in a variety of practical applications such as the manufacturing field, food processing industry, construction, agriculture, transportation, robotics, mechanical processing, information and communication technology, self-powered sources, Industry 4.0, forecasting techniques, drug safety, energy, and marine technology. It is hoped that triboelectric nanogenerator technology will be expanded for further practical applications in the near future.

## Figures and Tables

**Table 1:** Renewable electricity generation capacity by region from 2020 to 2024.

Region	Renewable Power Capacity (GW)					Renewable Share of the Electricity Capacity (%)					Growth in Comparison to 2020 (%)			
	2020	2021	2022	2023	2024	2020	2021	2022	2023	2024	2021	2022	2023	2024
Asia	1301.39	1456.18	1631.02	1960.96	2382.47	35.7	37.7	39.7	43.4	47.5	11.89	25.33	50.68	83.07
Europe	606.05	647.05	705.14	778.51	848.63	49.7	51.6	54.0	56.6	60.2	6.77	16.35	28.46	40.03
North America	424.73	462.21	492.47	527.11	573.01	30.6	32.3	33.7	35.2	37.3	8.82	15.95	24.10	34.91
South America	233.17	247.26	267.92	290.64	313.16	67.5	68.4	70.5	71.6	73.0	6.04	14.90	24.65	34.31
Eurasia	106.88	112.37	115.82	122.31	130.62	29.9	30.9	31.4	32.5	34.2	5.14	8.36	14.44	22.21
Oceania	47.04	52.35	58.78	65.11	73.78	44.9	47.4	50.2	53.3	56.0	11.29	24.96	38.41	56.85
Africa	53.68	55.58	59.29	62.67	66.89	22.3	22.6	23.5	24.4	25.4	3.54	10.45	16.75	24.61
Middle East	23.62	25.88	30.73	36.9	40.22	7.1	7.6	8.8	10.1	10.8	9.57	30.10	56.22	70.28
Central America and the Caribbean	16.43	17.06	17.61	18.67	19.27	35.7	37.2	38.1	38.7	38.7	3.83	7.18	13.63	17.29
Global Total	2812.98	3075.93	3378.79	3862.88	4448.05	36.6	38.4	40.3	43.1	46.4	9.35	20.11	37.32	58.13

**Table 3:** Comparison between TENGs and energy conversion technologies.

Items	TENG	EMG	PENG	Refs.
Materials	Triboelectric materials such as PTFE, PDMS, PEEK, PC, PP, PVDF, PA, PVC, FEP, PET, PEI, PS, PI, Nitrile, and PPS [227,228], biomaterial, cellulosic [229], organic materials [230], cadmium metal/cellulose composite [231], covalent organic [232], carbon nanomaterial, PVDF, CoNi [233], biopolymers [234], textiles [235], hydrogels [236], composite materials [237], fluorocarbon–graphene [238], rubber [239]	Magnetic materials [240], Fe_3_O_4_ [241], rubidium [242], dielectric genes [243], ZnO nanoparticle, TiO_2_, Ni-ZnO, MXene, In_2_O_3_ nanoparticle [244]	Piezoelectric materials [245] such as ceramics, single crystal of ZnO and CdS, polycrystal of BaTiO and PZT, polymers, and composites [246], LiNbO_3_, quartz, and PVDF [247], KNN, graphene [248], porous materials [249]	[227,228,229,230,231,232,233,234,235,236,237,238,239,240,241,242,243,244,245,246,247,248,249]
Working mechanism	The working mechanism of TENGs are related to mechanical energy–electricity conversion with operation modes including contact–separation type [250], sliding mode [251], free-standing mode [252], and single electrode mode [253]	EMG generates electric energy based on the electromagnetic induction principle that occurs as a magnetic flux in the closed circuit that generates the induced current under electromotive force induction [254]	PENG produces electricity based on the triboelectric effect as a material subjected to a mechanical pressure [255]	[250,251,252,253,254,255]
Input energy sources	TENG can convert vast mechanical energy into electricity such as mechanical energy [256], biomechanical energy [257], wind energy [258], wave energy [259], sound energy [260], vibration energy [261]	EMG can convert mechanical energy into electricity such as wave energy [262], kinetic energy [263], wind energy [264], motion, vibration [265]	PENG can transform mechanical energy into electricity such as mechanical stress [266], sound pressure [267], vibration [268]	[256,257,258,259,260,261,262,263,264,265,266,267,268]
Energy production	Producing electricity [269]	Generating electrical energy [270]	Producing electrical energy [271]	[269,270,271]
Advantages	Robust harvester [272], ability to work in extreme environments [273], light weight, high elasticity, high performance, high durability [274], renewable energy, clean energy [275], portability and flexibility [276]	Stability, high efficiency in energy conversion [277], low-input jigger frequency [278], renewable energy [279]	Some limitations that FENGs have been faced with include high precision, speed, and fast response [280], sonosensitizer [281]	[272,273,274,275,276]
Disadvantages	Some disadvantages that TENGs have been faced with include limitations in energy density, fluctuation in input signals, attachment of dusty [282], bulk size for TENG [282]	Some limitation that EMGs have been faced with such as low output voltage [283], power density limitation [284]	Some disadvantages that PENGs have been faced with include toxicity from metal particles and processing such as lead, electronic waste of piezoelectric materials, limitation of potential application by limiting temperature of piezoelectric material [285]	[282,283,284,285]
Potential applications	Smart active sensors [156], wearable sensors [286] self-powered wearable electronics [287], motion monitoring [288], e-skin technology [289], humidity sensors [290], intelligent sensors [291], ethanol sensing [292], monitoring structural health [293], deep learning applications [294], Internet of Things [295], human–machine [296], self-charging [297], self-powered sensor [298], aero-engine pipeline monitoring [299], clinical and healthcare [300], sports applications [301]	Some potential applications of EMGs include health monitoring [302], intelligent sensing [303], autonomous sensors [304], portable devices [305]	Harvesting energy, human healthcare [306], biomedical applications [307], harvesting energy, sensors [308], self-powered sensors [309], biomedical applications [310], sensors, precise instruments, health monitoring devices [311]	[156,286,287,288,289,290,291,292,293,294,295,296,297,298,299,300,301,302,303,304,305,306,307,308,309,310,311]
Integration ability	Alone Integrated into hybrid energy systems: TENG and electromagnetic nanogenerators [312], solar cells [313], piezoelectric nanogenerators in hybrid energy systems [314], TENG-EMG-PENG hybrid energy system for harvesting wind and vibration energies [315]	EMG integrated with TENG for harvesting energy and monitoring speed [316], a hybrid EMG and TENG system for harvesting wind energy [317], EMG-TENG hybrid system for harvesting wave energy [318]	PENG integrated with TENG for harvesting energy [319], PENG-TENG hybrid energy system for harvesting kinetic energy [320], PENG integrated with TENG and EMG to harvesting rotational energy [321]	[313,314,315,316,317,318,319,320,321,322],
Performance	Some TENGs have recently exhibited high performance such as a TENG with ultra-high current density with 8.75 A per square meter [323], a TENG with high output performance with an output voltage of 800 V [324], a TENG with a *V_OC_* of 420 V, Isc of 17 µA, and charge density of 1.3 µC [325], a TENG with an output power of 42.68 mW [326]	Some EMGs have recently generated electricity with peak output voltage of 86.4 V and peak current of 19.85 mA [327], an EMG with *V_OC_* of 8 V and Isc of 2.3 mA [325], an EMG with highest output power of 4.4 mW [326]	Some PENGs have recently shown good performance such as a PENG with an output voltage of 160 V [328], a PENG with power density of 28 µW/cm^2^ and *V_OC_* of 153 V [329], a PENG with a *V_OC_* of 14.59 V, Isc of 205.7 nA, and peak power density of about 7.5 mW/m^2^ [330]	[323,324,325,326,327,328,329,330]

## Data Availability

Data are presented in the coauthors’ research results and schematic drawings, which are available on request.

## Abbreviations

The following abbreviations are used in this manuscript:

TENG	Triboelectric nanogenerator
SD	Smart device
ET	Emerging technology
RE	Renewable energy
REEGC	Renewable energy generation capacity
GW	Gigawatt
IRENA	The International Renewable Energy Agency
IoT	Internet of Things
UAV	Unmanned aerial vehicle
5G-network	The fifth generation of mobile technology
°C	Degrees Celsius
*V_OC_*	Open-circuit voltage
σ	The triboelectric charge density
d	The distance between the two contact surfaces
$\varepsilon_{0}$	The vacuum permittivity
3D	Three dimensional
SDET	Smart devices and emerging technology
PV	Photovoltaic
ICT	Information and communication technology
BIM	Building information modeling
EMG	Electromagnetic generator
PENG	Piezoelectric nanogenerator
AI	Artificial intelligence
V	Voltage
PDMS	Polydimethylsiloxane
Al	Aluminum
PTFE	Polytetrafluoroethylene
Au	Gold
NaCl	Sodium chloride
PVC	Polyvinylchloride
Cu	Copper
H_2_	Hydro
PVDF	Polyvinylidene fluoride
PEEK	Polyether ether ketone
PC	Polycarbonate
PP	Polypropylene
PA	Nylon
FEP	Fluorinated ethylene propylene
PET	Polyethylene terephthalate
PEI	Polyetherimide
PS	Polystyrene
PI	Polyimide
PPS	Polyphenylenesulphide
CoNi	Cobalt–Nickel
PZT	Zirconate titanate
BaTiO_3_	Barium titanate
LiNbO_3_	Lithium niobate
ZnO	Zinc oxide
KNN	Potassium sodium niobate
Cu_2_Te	Copper telluride
A	Ampe
HMI	Human–machine interface
W/m^2^	Watt per square meter
µW	Microwatt
mW	Milliwatt
W	Watt
ANN	Artificial neural network
PMM	Power management module
